# An Integrative Approach to Assess the Environmental Impacts of Gold Mining Contamination in the Amazon

**DOI:** 10.3390/toxics9070149

**Published:** 2021-06-26

**Authors:** Mariana V. Capparelli, Marcela Cabrera, Andreu Rico, Oscar Lucas-Solis, Daniela Alvear-S, Samantha Vasco, Emily Galarza, Lady Shiguango, Veronica Pinos-Velez, Andrés Pérez-González, Rodrigo Espinosa, Gabriel M. Moulatlet

**Affiliations:** 1 Facultad de Ciencias de La Tierra y Agua, Universidad Regional Amazónica Ikiam, Tena 150150, Ecuador; marivcap@gmail.com (M.V.C.); marcela.cabrera@ikiam.edu.ec (M.C.); oscar.lucas@est.ikiam.edu.ec (O.L.-S.); cyntia.alvear@est.ikiam.edu.ec (D.A.-S.); emily.galarza@est.ikiam.edu.ec (E.G.); lady.shiguango@est.ikiam.edu.ec (L.S.); 2 Instituto de Ciencias del Mar y Limnología-Estación El Carmen, Universidad Nacional Autónoma de México, Ciudad del Carmen 24157, Mexico; 3 Laboratorio Nacional de Referencia Del Agua, Universidad Regional Amazónica Ikiam, Tena 150150, Ecuador; 4 IMDEA Water Institute, Science and Technology Campus of the University of Alcalá, 28805 Alcalá de Henares, Spain; andreu.rico@imdea.org; 5 Cavanilles Institute of Biodiversity and Evolutionary Biology, University of Valencia, 46980 Paterna, Spain; 6 Facultad de Ciencias de La Vida, Universidad Regional Amazónica Ikiam, Tena 150150, Ecuador; samantha.vasco@est.ikiam.edu.ec (S.V.); rodrigo.espinosa@ikiam.edu.ec (R.E.); 7 Departamento de Recursos Hídricos y Ciencias Ambientales, Facultad de Ciencias Químicas, Universidad de Cuenca, Cuenca 010202, Ecuador; veronica.pinos@ucuenca.edu.ec; 8 Departamento de Biociencias, Facultad de Ciencias Químicas, Universidad de Cuenca, Cuenca 010202, Ecuador; 9 Grupo de Investigación en Quimiometría y QSAR, Facultad de Ciencia y Tecnología, Universidad del Azuay, Cuenca 010204, Ecuador; aperez@uazuay.edu.ec

**Keywords:** metals, environmental monitoring, bioassays, Amazon River, Amazonia, mining

## Abstract

As the number of legal and illegal gold mining sites increases in the Andes–Amazonia region, integrative methods to evaluate the effects of mining pollution on freshwater ecosystems are of paramount importance. Here, we sampled water and sediments in 11 sites potentially affected by gold mining activities in the Napo province (Ecuador). The environmental impacts were evaluated using the following lines of evidence (LOEs): water physicochemical parameters, metal exposure concentrations, macroinvertebrate community response (AAMBI), and toxicity by conducting bioassays with *Lactuca sativa* and *Daphnia magna*. Dissolved oxygen and total suspended solids were under (<80%) and above (>130 mg/Ls) quality standards 65% of the sites. Ag, Al, As, Cd, Cu, Fe, Mn, Pb, and Zn in water and V, B, and Cr in sediments were detected above quality standards at sampled sites. Nine out of eleven sites were classified as having bad environmental quality based on the AAMBI. *L. sativa* seed germination in both water (37% to 70%) and sediment (0% to 65%) indicate significant toxicity. In five sites, neonates of *D. magna* showed a 25% reduction in survival compared to the control. Our integrated LOEs index ranked sites regarding their environmental degradation. We recommend environmental impact monitoring of the mining expansion at the Andes–Amazonia region using multiple LOEs.

## 1. Introduction

The Amazon basin has been historically impacted by mining activities [1,2,3]. Mineral soils underlying the Amazon forests contain elevated levels of gold, whose exploitation results in intensive land-use modifications [4] and freshwater contamination with mining tailings [5]. Mining areas are expanding in protected and unprotected areas of the Amazon due to governmental incentives [6,7,8]. Moreover, illegal mining has recently expanded in several parts of the Amazon basin, which has resulted in an environmental impact whose extension is difficult to ascertain [4,9].

Between 2008 and 2009, the Ecuadorian government redefined policies to promote the growth of the mining sector [10]. During the 2008–2018 period, mining exports increased by 86%, positioning it as the fourth product of exportation in Ecuador [11]. Given the importance of gold mining to Ecuador’s economy and the proliferation of mining concessions, information on the environmental impacts caused by this activity is crucial. The ecosystems on the eastern Andes of Ecuador, at the transitions with Amazonia, have been largely impacted by gold mining activities [12,13]. In the last decade, new gold mining concessions have been approved in the Napo province [8]. Until 2020, 152 concessions were registered at the national mining cadastre [14], most of them located along the Anzu and Jatunyacu rivers, where alluvial gold mining predominates. About 60% of the concessions were authorized for artisanal mining, leaving the remaining ones for industrial or medium-scale mining. The mining scale is defined by extension, the processed volume of material, and the degree of financial investments [10,14]. Even though artisanal small-scale gold mining (i.e., mining without heavy machinery and granted to small companies or local communities) is the most common authorized concession, industrial gold mining represents 98% of the total territory for the exploitation of gold in the Napo province (33,718 ha).

To effectively assess the ecological impacts caused by gold mining activities, integrative studies that involve different lines of evidence (LOEs), such as geochemical assessments, ecotoxicological tests, and biological monitoring, have been proposed [15]. While physicochemical and metal exposure analyses provide information on the kind and degree of the contamination, ecotoxicological tests and bioassays are used to describe the potential biological effects of the measured contamination levels on sentinel species [16,17]. In addition, biological monitoring evaluates structural changes in the ecosystem and identifies sensitive species to different pollution levels. The integration of these multiple LOEs has been traditionally used in the risk assessment of contaminated sites to elucidate cause–effect relationships and allows the characterization of sampling sites regarding the nature and magnitude of the ecological impact [17,18,19].

Some studies have characterized the environmental concentrations of metals and metalloids (hereafter referred to as metals) in freshwater ecosystems of the Andean–Amazon region impacted by mining activities, indicating the exceedance of national and international quality standards [12,20]. However, the impacts of these metals on relevant biological endpoints for the Andean–Amazon region have not been evaluated. This is of utmost importance given the relevance of this region for maintaining the hydrological dynamics of the Amazon basin and several ecosystem services, including water and food source provision for local communities and biodiversity preservation [21,22]. For instance, the rivers of the Napo basin drain from the steep Andean slopes towards Amazonian lowlands, so it is expected that mining impacts along the river course can extend to the lower parts of the Amazon basin.

The aim of this study was to characterize the environmental concentrations of metals in selected areas of the Napo region and to evaluate their impacts on freshwater ecosystems by providing an ecosystem impact classification ranking based on several LOEs, including: (1) water physicochemical parameters, (2) metal exposure in water and sediments, (3) benthic macroinvertebrates, and (4) bioassays performed with model plant and invertebrate species. Through this study, we characterize the magnitude and spread of the contamination posed by medium-scale to industrial-scale gold metal mining in the Ecuadorian Andes–Amazonia region and provide a method that can be extrapolated to other regions to assess the degree of environmental degradation.

## 2. Materials and Methods

### 2.1. Study Area and Sampling

Our study focused on the mining conducted at the tributaries of the Anzu, Jatunyacu, and Napo rivers (the latter being formed by the union of the two previous ones). These three rivers comprise about 90% of the gold mining concessions in the Napo province. The Napo province has precipitation rates above 4000 mm/y. In addition to the great biodiversity of flora and fauna, the study area is known to be a geodiversity hotspot, given the content of high-value minerals, such as gold. Gold alluvial deposits of the types of placer and paleoplacer predominate in the area.

**Figure 1:**
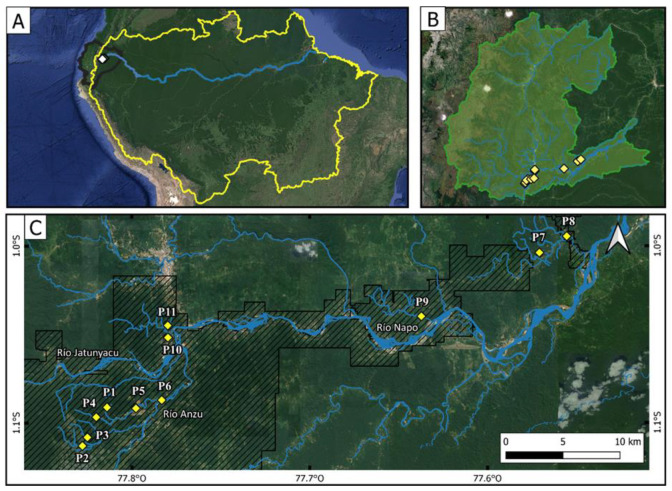
(**A**) Location of the study area in the Amazon basin. (**B**) Napo province of Ecuador and location of the sampling sites (yellow diamonds). (**C**) Detailed distribution of the sampling sites (yellow diamonds). Dashed polygons show the total area under concession for gold mining activities according to [14].

Eleven sites located along rivers directly affected by medium-scale to industrial-scale gold mining were sampled in December of 2020 (Figure 1; Table S1 of Supplementary Materials). Precipitation above 1 mm was not registered the day before sampling (Meteorological Station of Ikiam University, http://meteorologia.ikiam.edu.ec:3838/meteoviewer/ (accessed on 17 November 2020)). All sites were located within mining concession territories. Surface water (100 mL) and surface sediment (150 g) samples were taken. Water samples were filtered and then acidified (to pH 2) in the field with HCl for metal analysis. After collection, water and sediment samples were stored at 4 °C for no longer than 1 month until processing. Additionally, water samples (1 L) were collected in plastic bottles and stored at 4 °C for performing bioassays. Macroinvertebrates were collected using a D-shaped net, according to the methods described by [23]. For this, the benthic substrate was disturbed for 5 min in riversides, ponds, under the rocks, and leaf packs to collect all representative organisms. The macroinvertebrate samples were stored in glass flasks with 96% alcohol and then transported to the laboratory for organism counting and identification.

### 2.2. Measurements of Physicochemical Parameters

Conductivity, pH, ORP, total dissolved solids (TDS), and dissolved oxygen (DO) were measured in situ using a professional plus multiparameter. Turbidity was measured in situ with a HACH 2100 Q turbidimeter. The equipment was previously calibrated with standard solutions. In the laboratory, dissolved organic carbon (DOC) concentrations were measured using a total organic carbon analyzer (TOC-L Shimadzu, Kyoto, Japan). The total suspended solids (TSS) were analyzed according to [24]. Water hardness was determined according to [25].

### 2.3. Metal Analyses

Dissolved metal analyses in the water and sediment samples were performed at the Laboratory of the University of Cuenca (Ecuador) after acid digestion using 8 mL of ultrapure nitric acid and 2 mL of hydrochloric acid (Merck trend). The samples were analyzed in triplicate using a Perkin Elmer 350X ICP-MS (Walthan, MA, USA). The standard method was used for 44 metals, both in water and sediments. For the reading of metallic and nonmetallic analytes, an adaptation of Method 200.8 EPA (United States Environmental Protection Agency) was used [26]. Calibration curves were created from a multielement standard Inorganic Venture, at concentrations from 0.1 to 0.0005 mg L^−1^. Quality control for major and trace elements analysis was implemented using certified reference water (CRM 1640a) and sediment (CRM 1646a) (NIST, Gaithersburg, MD, USA) every 10 samples, as well as at the beginning and at the end of each sample batch. Recovery percentages were calculated to determine possible matrix effects and method accuracy. All major and trace metal concentrations were corrected based on the recovery percentages obtained in each analysis, which ranged from 91% to 100% for water and 69% to 93% for sediments.

### 2.4. Calculation of the Andean–Amazon Biotic Index for Macroinvertebrates

All macroinvertebrates were classified and identified taxonomically to the family level using specialized taxonomic keys [23,27]. Then, the Andean–Amazon Biotic Index (AAMBI) [28] was calculated for each sampling site. This index assigns a numerical value to each macroinvertebrate family, which goes from 1 for contamination-tolerant families to 10 for highly susceptible ones. The sum of these numerical values is classified into five water quality categories: excellent (>121), very good (90–120), good (50–89), regular (36–49), and bad (<35).

### 2.5. Bioassays

Bioassays with *Daphnia magna* and *Lactuca sativa* were performed at the National Reference Laboratory for Water of the Universidad Regional Amazónica Ikiam (Ecuador). Ten neonates of *D. magna* of 24 h of a parthenogenetic culture (4th generation) were exposed to 10 mL of water from each sampling site and standard culture medium as a control [29], using two replicates. After 48 h, the survival rate was calculated. The *L. sativa* tests were performed by exposing seeds to the collected water and sediment samples according to [12] and [19]. 2.5 mL volume of the water samples and control (distilled water) was transferred in duplicate to filter papers in Petri dishes. Then, 15 *L. sativa* seeds were evenly distributed on fully moistened filter papers. Petri dishes were covered with aluminum foil and allowed to stand for 5 days in the dark at room temperature. For sediment samples, 15 seeds were evenly distributed in 10 g of sediment in a plastic container (100 mL). The sediment used as a control was taken from an area far from any source of contamination. The containers were then incubated at 25 °C in the dark for 24 h and maintained under a 12 h/12 h (light/dark) photoperiod for 14 days. After that, germination rates and root elongation of the germinated seedlings were measured.

### 2.6. Data Analyses

Metal concentrations in water samples were compared to the environmental quality standards established by the Ecuadorian legislation [30], the United States Environmental Protection Agency [31], and the Canadian Environmental Quality Guidelines [32]. As for sediment samples, the CCME (2002) environmental quality standard was used. Measured concentrations were compared to the threshold effect level (TEL), which represents the concentration below which rare adverse biological effects are expected, and to the probable effect level (PEL), which defines the level above which adverse effects are frequently expected to occur [33].

Statistically significant differences in the survival rate of *D. magna* in the control and in the collected water samples were assessed using the Student’s *t* test. The assessment endpoints in the *L. sativa* tests were the germination percentage and the average length of the root of the germinated seedlings. Statistically significant differences between the water and sediment controls and the collected samples for the *L. sativa* endpoints were also evaluated with the Student’s *t* test. Prior to that, the normality and homoscedasticity of the root length data were evaluated using the Shapiro–Wilks and Fligner tests, respectively. Samples were considered toxic or eutrophic when the mean length of the seedlings was significantly lower or higher than the control, respectively. Statistically significant differences were assumed when the calculated *p*-value was ≤ 0.05.

The integration of the four LOEs (i.e., physicochemical parameters, metal exposure, biological monitoring, bioassays) was carried out using an integrated quantitative index that uses the complete decision matrix for the four LOEs [19]. We assigned seven classes of normalized values from 0 to 5 to each of the LOEs depending on multiple criteria (Table S2, Supplementary Materials). The sum of the assigned scores to each site can be interpreted as the degree of environmental impact, where the maximum index value is 20 (i.e., no observed degradation), and the minimum index value is 0 (i.e., full degradation).

Principal component analysis (PCA) was used to explore the relationship between the physicochemical parameters, metal concentrations, the AAMBI values, and the results of the toxicity bioassays in the water and sediments samples. Only the metals Ag, Al, As, Cd, Cu, Fe, K, Mg, Na, Ni, Mn, Pb, and Zn and the parameters TSS, DO, color, and conductivity were retained in this analysis. All variables were normalized by site, by setting the sum of squares equal to 1. The first two principal components (PCs) were investigated, and their correlations to each variable were tested through Pearson’s correlation test.

Hierarchical cluster analyses were used to assess the presence of natural clusters among sampling sites by an iterative process that defined clusters based on the dissimilarities of two sites. Dissimilarities between sites were calculated by Euclidean distances for normalized variables (variable values divided by total sum of each variable); the Group Average Link was used as the agglomeration method in the classification. All statistical analyses were performed using the R software [34].

## 3. Results

### 3.1. Physicochemical Parameters

DO (except for sites P1 and P2) and TSS (except for Sites P2, P4, P7, and P8) were above the thresholds set by TULSMA, CCME, and EPA (<80% Sat and >130 mg L^−1^, respectively). pH values ranged from 6.5 to 8.0; water temperature was between 25 and 30 °C; turbidity (NTU) ranged from 10 to 1690; conductivity ranged from 25 to 187 μs cm^−1^; color ranged from 70 to 8500 Pt-Co; DOC ranged from 1.8 to 4 mg L^−1^; and ORP ranged from 77 to 174 MV (Table 1).

### 3.2. Metal Concentrations

**Figure 2:**
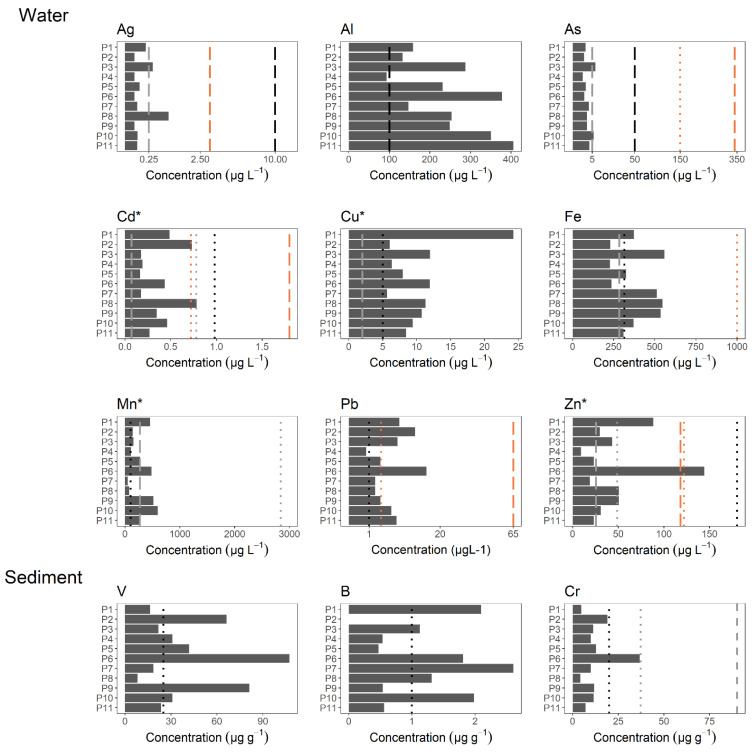
Average metal concentrations in water (μg L^−1^) and in sediment samples (μg g^−1^). Only metals that were detected above permissible limits are shown. For water samples, vertical lines indicate the limits established by the Canadian Environmental Quality Guidelines for the Protection of Aquatic Life [32] for short- (gray dotted lines) and long-term effects (gray dashed lines), and the Ecuadorian legislation TULSMA (black dotted lines) and Water Quality Criteria for the Protection of Aquatic Life [31] for acute (brown dashed lines) and chronic (brown dotted lines) effects. For sediment samples, vertical lines indicate the limits established by the Ecuadorian legislation [30] (black dotted lines) and the threshold effect level (TEL; gray dotted lines) and the probable effect level (PEL; gray dashed lines), as defined by CCME (2002). * The highest CCME short- or long-term thresholds are shown. Actual threshold values can be found in the Table S3 of the Supplementary Materials.

Water quality standards were exceeded by at least one of the following metals in all sites: Ag, Al, As, Cd, Cu, Fe, Mn, and Pb (Figure 2). Sites with the highest number of metals exceeding the quality standards were P3, P6, P10, and P11. Cd was above quality standards for all sites, and in Sites P2 and P6, it was detected above the limit for chronic contamination. Pb was above the limit for chronic contamination in 55% of the sites. For Zn, Site P6 exceeded more than 10 times-fold the limits for acute and chronic contamination. Sediment quality standards were exceeded by V and B in more than 55% of the sites; Cr was detected above limits in P6.

### 3.3. Macroinvertebrates

**Figure 3:**
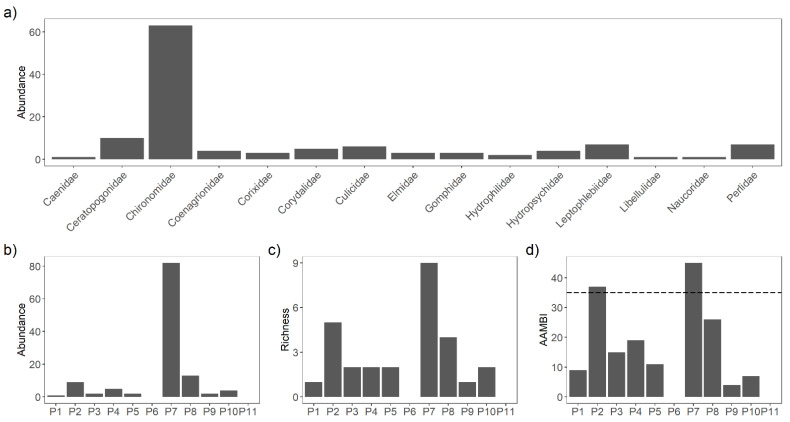
Results of the macroinvertebrate sampling: (**a**) abundance of individuals as reported in each macroinvertebrate family; (**b**) total abundance of individuals in each sampling site; (**c**) family richness in each sampling site; (**d**) calculated AAMBI values for each site. The dashed line indicates the AAMBI value (35) below which sampling sites are classified as “bad” quality.

Chironomidae was the most abundant macroinvertebrate family (Figure 3a). Site P7 had the highest abundance of individuals (76), while at Sites P6 and P11, no macroinvertebrates were found (Figure 3b). Family richness was higher in Sites P2 and P7 (Figure 3c) The evaluation of water quality based on the AAMBI index (Figure 3d) showed that 63% of the sites had scores below the lowest AAMBI classification (< 35), indicating poor water quality.

### 3.4. Toxicity Bioassays

**Figure 4:**
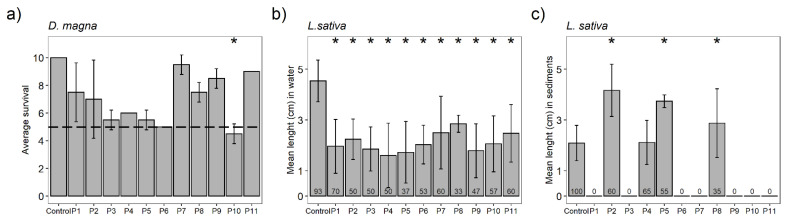
Results of the bioassays performed with neonates of *Daphnia magna* (**a**) and seeds of *Lactuca sativa* (**b**,**c**) with water and sediment samples. For the *Daphnia magna* tests, data show the average number of surviving neonates (48 h) ± standard deviation, and the asterisk (*) represents significant differences (*p* ≤ 0.05) with respect to control. The dashed black line indicates a 50% reduction in survival. For the *L. sativa* tests, data represent the average length ± standard deviation. (*) represents significant differences (*p* ≤ 0.05) with respect to the control, and the numbers within the bars show the germination percentages (*n* = 15).

*D. magna* neonates showed more than 25% reduction in survival as compared to the experimental control (Figure 4a) in Sites P3, P4, P5, and P6. Site P10 was the only one where a significant difference from the control (*p* ≤ 0.05) was detected, indicating clear sample toxicity.

*L. sativa* seed germination rates were 37–70% in the water samples and 90% in the experimental control. Regarding water phytotoxicity (Figure 4b), seeds from all sampling sites inhibited root growth. Regarding sediment phytotoxicity (Figure 4c), seeds from Sites P2 (60%, germination), P5 (55%, germination), and P8 (35%, germination) displayed enhanced root growth, which may indicate hormesis response. Seeds did not germinate in 7 out of the 11 samples, while germination of the control was 100%.

### 3.5. Integrative Analysis of the Four LOEs

The different LOEs were used to rank the different sampling sites according to the level of impact or degradation. Our results showed that the P7 site had the highest score (13.77), followed by P2 (12.90), indicating the lowest ecological impact compared to the other sites. On the other hand, Sites P11 (10.68), P10 (10.26), and P6 (10.34) were the most affected by gold mining (Table 2).

### 3.6. PCA and Hierarchical Cluster Analysis

**Figure 5:**
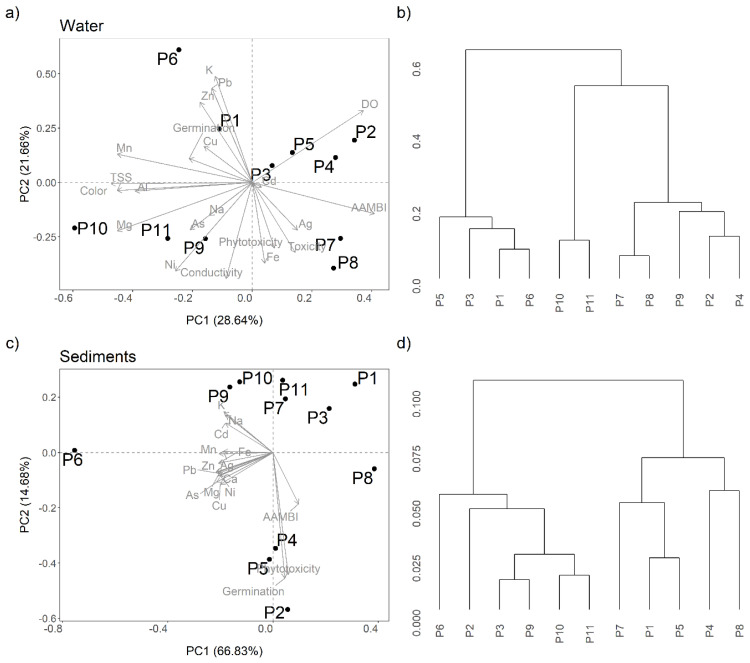
(**a**,**c**) PCA analysis for the water (**a**) and sediment (**c**) samples collected in Ecuadorian Amazon rivers affected by gold mining. The dimensional space is determined by the two first PCA axes. (**b**,**d**) Results of the hierarchical cluster analysis for water (**b**) and sediments (**d**). The description of the sampling sites is provided in the Table S1 of the Supplementary Materials.

For water samples (Figure 5a), the PC1 explained 28% and PC2 explained 21% of data variance. Sites P2, P4, and P5 were highly correlated with DO concentrations. P6 was correlated with metal contamination, while phytotoxicity and *D. magna* toxicity were related to Site P8. Sampling sites that were geographically closer to each other were found to be in the same cluster, such as P5 and P6, P7 and P8, or P10 and P11 (Figure 5b). For sediment, PC1 explained 66.83% of data variance and separated the samples mostly by metal concentrations, while PC2 (14.68% of data variance) separated sites by physicochemical parameters and phytotoxicity (Figure 5c). P6 was the site mostly associated with metal contamination. The third PC apparently joined residual variances. Three sample clusters could be identified at the cut-off value of 0.075 (Figure 5d).

## 4. Discussion

The integrative assessment carried out in this study, combining multiple LOEs, demonstrates that medium-scale to industrial-scale gold mining has a broad impact on the quality of freshwater ecosystems in the Napo River network. The identified impacts varied regarding their nature and magnitude in the different sampling sites. Overall, metals were considered relevant stressors, with environmental concentrations exceeding quality standards for both water and sediments in all sites. Exceedance of regulatory thresholds has been reported in several areas affected by mining exploitation in the Amazon basin [12,35,36,37]. Excessive metal concentrations in water and sediments, both those considered essential and nonessential to biological systems, can cause serious damage to aquatic life by affecting the reproductive physiology of fish and invertebrates, inducing carcinogenicity, genotoxicity, and causing adverse effects on the endocrine systems, such as liver necrosis and ultimately death [37,38].

The highest macroinvertebrate AAMBI index score measured in this study was 40, while in areas with little or no mining impact, other authors have reported values above 80 [19,39]. Sites P2 and P7 were classified as having the best water quality according to the AAMBI classification. All the others presented poor water quality according to the AAMBI, and some (P6 and P11) even denoted a full absence of macroinvertebrates, which can be related to the high concentrations of metals found in these sites. The absence of macroinvertebrates was reported in a sampling conducted 8 months earlier at Site P6 (Site “GM”) by [19]. The prolonged absence of macroinvertebrates indicates an elevated degree of environmental disturbance and can also be interpreted as a warning indicator for pervasive local mining effects. Our results confirmed that gold-mining-derived water and sediment contamination have direct negative effects on benthic invertebrate communities, reducing species abundance and richness and modifying the community structure [40]. Moreover, all the sites classified as having moderate to high impact had full absence or low abundance of most macroinvertebrate taxa, with the exception of the family Chironomidae. This is consistent with other studies that showed a predominance of chironomids in sites exposed to metal contamination [41].

The alteration of physicochemical parameters TSS, DO, turbidity, and color indicates that there is a constant load of potentially metal-contaminated sediments into the rivers. An associated impact of gold mining is the earth movement made by heavy machinery on river margins, causing the erosion of the riverbanks and intense modifications to the landscape. Moreover, to dig waste pools, mining machines move large amounts of soil onto sediment piles, which are subjected to erosions caused by the intensive rainfall in the area. Our sampling included sites where mining was active during the time of our sampling (P1, P3, P9) and some that received mining tailings transported by the rivers (P6, P10, P11). At all these sites, DO concentrations were found to be below permitted levels and were slightly lower than those reported in a previous monitoring study [19], which may indicate an increase in mining activity and a worsening of the water quality status in the study area. Moreover, extremely high TSS concentrations were also reported for these sites. High TSS are associated with the reduction of light penetration, reduction of primary productivity, and a decrease of DO in water [42]. TSS are also potential carriers of metals to the rivers, which can negatively affect some macroinvertebrate groups (e.g., filter and deposit feeders). In all sites in which high phytotoxicity was registered, both low AAMBI values and high mortality of *D. magna* were also reported. This suggests that the toxic effects of gold mining contamination can affect multiple components of the ecosystem, from primary producers to consumers, and indirectly affects habitat quality and the availability of food resources for predators. Thus, as also suggested in previous studies [43], the alteration of water physicochemical parameters by mining (by tailings and metal remobilization and enrichment) can be detrimental to the local freshwater biodiversity.

In 63% of the sites, no germination of *L. sativa* seeds was reported, suggesting high toxicity.. This result confirms the findings of other studies describing the phytotoxicity caused by mining pollution in *L. sativa* [12,19,44] and studies reporting growth inhibition associated with Cu, Pb, Zn, Ni, and Cd exposure [44,45,46]. On the other hand, contaminants may also stimulate *L. sativa* seed growth by generating stimulatory responses [47,48], as observed in the sediment samples of Sites P2, P5, and P8. The hormesis response phenomenon, characterized by low-dose stimulation and a high-dose inhibition [49,50], can be observed in a wide range of biological endpoints. At higher metal concentrations, *L. sativa* seed germination inhibition would be expected, while growth stimulation may occur at low metal concentrations. As denoted in this study, gold mining activity can induce water and sediment phytotoxicity, due to high toxic trace element concentrations (e.g., Cd, Hg, and Cu) and the presence of soluble salts [51]. However, the degree of toxicity of these metals depends on physicochemical parameters that induce metal speciation, such as pH, oxide-reduction potential, hardness, and temperature [52]. Metals tend to become more reactive at lower pH, higher oxidation potential, lower hardness, and higher temperature [53]. Most of the pH values found in this study were above permitted thresholds (Table 1), which indicates that some elements may be poorly soluble or present in less reactive forms, associated with hydroxides, oxides, carbonates, and silicates.

Our multiple LOEs integrated index indicated that all monitored sites suffered a notable degradation level. Sites P2 and P7 were the ones showing the highest rankings for all evaluated LOEs. Although both sites are located upriver from mining concessions, they still present signs of chronical contamination due to historical mining activities. The high level of degradation of these study sites can be attributed to their location at basin outlets, where mining sediments and mining tailings get immediately transported downstream and deposited. River meanders, floodplains, and basin outlets can be considered as sink areas that constantly receive washed-off materials from upstream areas. The flooding regimes of the rivers of the upper Napo basin may also lead to the storage of contaminated sediments on river margins, which can be eroded and sporadically redistributed [54,55]. Whether this is the case in the study area, requires further investigations. However, it is clear from our investigation that the most contaminated sites were located at basin outlets downstream from mining camps that have received significant transportation and sedimentation of mining disposals.

In addition to the ecosystem impacts, potential social impacts caused by mining activities should be further investigated. For instance, Sites P6 and P11 are located less than 1 km away from indigenous communities. The degree of contamination of both water and sediments in these sites turns the water and food sources not recommended for human consumption, as pointed out by [19] in their sampling performed two years before ours. Therefore, it could be that local communities have been consuming water and fish with metal levels above the recommended for a relatively long period already. Further examination on the temporal and spatial extension of contamination and their impacts on the health status of local indigenous communities is recommended.

## 5. Conclusions

Establishing causal relationships between chemical monitoring data and ecosystem effects is challenging given the number of stressors that co-occur in freshwater ecosystems and their direct and indirect impacts on biodiversity. The multiple LOEs approach described here can be used to classify areas regarding their level of environmental degradation and to characterize the different drivers that impact freshwater ecosystems. Likewise, it can help to establish conservation and restoration objectives, aiding in the development of environmental management plans for mining areas in the Amazon region. Based on the results of our study, we recommend that further monitoring focuses on selected basin outlets, as contamination accumulates in those areas, and that macroinvertebrates, DO, TSS, Cu, Pb, Zn, Ni, and Cd are periodically monitored as indicators of ecosystem deterioration. Given the importance of the Andes–Amazonia region for biodiversity preservation and ecosystem service provision, we also recommend further control of the gold mining expansion and its continued environmental monitoring using multiple LOEs.

## Figures and Tables

**Table 1:** Physicochemical parameters measured in the different sampling sites (see Figure 1 for site location). Values highlighted in bold are above (TSS) or below (DO) the thresholds for the Water Quality Criteria for the Protection of Aquatic Life [31], the Ecuadorian Guidelines [30], or the Canadian Environmental Quality Guidelines [32].

Sites	Physicochemical Parameters									
	pH	T (°C)	Turbidity (NTU)	TDS (mg L^−1^)	TSS (mg L^−1^)	Conductivity (μs/cm)	DO (% sat)	Color (Pt-Co)	DOC (mg L^−1^)	Hardness (mg CaCO_3_ L^−1^)
P1	6.91	23.3	765	45.5	**698**	67.6	80.6	2800	4.31	11.8
P2	6.7	25.6	10.2	16.25	3	25.3	81.5	82	1.85	3.9
P3	6.55	30	277	14.95	**523**	25	**75**	375	2.4	5.3
P4	6.67	28.3	24	33.2	19	53.8	**76.2**	63	3.86	7.6
P5	7.17	31	246	31.2	**171**	52.1	**78.4**	1200	2.98	9.0
P6	6.8	29.2	1457	27.3	**953**	45.8	**76.6**	3950	3.52	12.3
P7	6.61	30	37.3	42.2	19	70.9	**74.7**	245	4.07	37.9
P8	8.06	28.1	28.2	115	6	187.2	**76.9**	78	1.89	34.5
P9	7.18	26.5	339	96.2	**201**	152.3	**56.5**	650	1.74	23.4
P10	7.37	25.1	5026	57.85	**3200**	88.6	**54.5**	8500	2.27	37.4
P11	7.79	26.7	1690	79.95	**1024**	127.3	**50.8**	5250	2.39	30.6
CCME	6.5–8.5	22.5–27.5	-	500	-	500	>80	-	-	-
TULSMA	6.5–9.0	22.0–28.0	10	1000	130	1000	>80	-	-	-
US EPA	6.5–9.0	22.0–28.0	-	500	-	500	>80	-	-	-

**Table 2:** Integrative matrix analysis of four LOEs: physicochemical parameters, metal concentrations, toxicity assessment with bioassays, and macroinvertebrate monitoring with the Andean–Amazon Biotic Index (AAMBI). Sites are ranked from low to high levels of environmental degradation.

Sites	Physicochemical Parameters	Metal Concentrations	Toxicity (Bioassays)	Macroinvertebrates (AAMBI)	Total
P7	4.01	4.12	3.84	1.8	13.77
P2	4.25	4.08	3.09	1.48	12.90
P8	4.03	4.06	3.55	1.04	12.68
P4	4.38	4.56	2.5	0.76	12.20
P9	3.84	4.09	3.79	0.16	11.88
P1	3.84	4.17	3.06	0.36	11.43
P3	3.71	4.18	2.49	0.6	10.98
P5	3.87	4.15	2.43	0.44	10.89
P11	3.82	4.29	2.57	0	10.68
P6	3.45	3.74	3.15	0	10.34
P10	3.67	3.96	2.35	0.28	10.26

## Data Availability

Not applicable.

## Supplementary Materials

The following are available online at https://www.mdpi.com/article/10.3390/toxics9070149/s1, Table S1: Geographic coordinates and description of collection sites. Depth at the center of the main river channel and width at the sampling location are reported, Table S2: Parameters and the respective scores used to calculate the integrated index that includes the four LOEs of freshwater parameters (physicochemical, metal concentrations, macroinvertebrates, and toxicity) assessed in the study area. Table S3: Actual threshold values of CCME short- and long-term values calculated for the metals Cd, Zn, Mn and Cu. Calculations are based on pH, DOC and Hardness, as indicated in the equations found at the CCME Canadian Environmental Quality Guidelines (Vol. 2) 2002.
