# Supplementary material for: An Integrative Approach to Assess the Environmental Impacts of Gold Mining Contamination in the Amazon
Source: Toxics. 2021 Jun 26;9(7):149. doi: 10.3390/toxics9070149 (PMC8309824; doi:10.3390/toxics9070149)
Supplement: Supplementary file 1 [file toxics-09-00149-s001.zip › toxics-1242579-supplementary.pdf]

toxics

Article

Supplementary Material: An Integrative Approach to Assess the Environmental Impacts of Gold Mining Contamination in the Amazon

Mariana V. Capparelli, Marcela Cabrera, Andreu Rico, Oscar Lucas-Solis, Daniela Alvear-S, Samantha Vasco, Emily Galarza, Lady Shiguango, Veronica Pinos-Velez, Andrés Pérez-González, Rodrigo Espinosa and Gabriel M. Moulatlet

Table S1. Geographic coordinates and description of collection sites. Depth at the center of the main river channel and width at the sampling location are reported.

Sites	Coordinates	Elevation (m)	River			Local Description
			Name	Depth (m)	Width (m)	
P1	1°06′27″ S 77°51′35″ W	603.8	Río Chumbiyacu	0.26	2.4	P1 is in the Moretecocha mining area, within a forest area. The stream receives the wastewater from the waste pools. The mining machines were in operation during the sampling.
P2	1°08′17″ S 77°52′44″ W	577.9	Río Chucapi	0.12	6	P2 is in a river that drains to an abandoned mining area. There was secondary vegetation on the banks of the river.
P3	1°08′11″ S 77°52′41″ W	586.5	Río Chucapi	0.08	0.6	P3 is located within an active mining area. The stream receives the wastewater from the waste pools. The mining machines were in operation during the sampling.
P4	1°06′55″ S 77°52′05″ W	580.0	Río Chumbiyacu	0.34	4	P4 is in an area highly affected by the opening of a road. Construction machines were crossing the river and moving sediments from river margins and riverbed.
P5	1°06′33″ S 77°50′10″ W	502.4	Río Chumbiyacu	0.33	6	P5 is in an abandoned mining area. Presence of secondary vegetation. The river receives water from mining areas.
P6	1°06′7″ S 77°49′00″ W	465.2	Río Chumbiyacu	0.19	14	P6 is located near Chumbiyacu River bridge, downstream from point P5.

P7	0°59′13″ S 77°31′01″ W	360.9	Río Huambuno	0.45	0.5	P7 is located about 50 m upstream of an active mining area.
P8	0°58′20″ S 77°29′51″ W	355.8	Río Huambuno	0.57	15	P8 is situated near Huambuno River bridge, downstream from point P7.
P9	1°02′09″ S 77°36′43″ W	440.2	Río Tuyano	0.17	0.15	P9 is located within an active mining zone. The mining machines were in operation during the sampling. The riverbed was totally modified for the benefit of filling waste pools and washing alluvial sediments.
P10	1°03′01″ S 77°48′42″ W	441.6	Quebrada Yutzupino	0.02	0.63	P10 is located about 60 m from the Portoviejo highway, close to Puerto Napo. Downstream from a mining area.
P11	1°03′00″ S 77°48′42″ W	440.4	Río Yutzupino	0.26	0.04	P11 is located about 40 m from the Portoviejo highway, close to Puerto Napo. Downstream from a mining area.

Integrative index based on Multiple Lines of Evidence

For the LOE physicochemical and the LOE metals exposure assessment, two equal methods of evaluation were used to assign values into the integrated index (Table S2). First, we select only the physicochemical parameters and metal species above TULSMA permissible limits. Then, we determine classes of values depending on how much these parameters exceed permissible limits on a 50 times-base, i.e., if values were 50 times lower than permissible limits, they received the highest score (low environment impact); if values were 50 times or more above the limits, scores were lower (high environmental impact). Secondly, we established a measure based on the total number of physicochemical parameters and metal species that exceed the permissible limits for each site. This measure is complementary to the first evaluation method because it is independent of the concentration or value of each parameter. In this case, if less than 20% of the physicochemical parameters and metals exceed the permissible limit, the higher score is assigned; if more than 80% of the physico-chemical parameters and metals species exceed the permissible limit, the lower score is assigned to the site.

For the LOE phytotoxicity of *L. sativa*, whenever growth enhanced or inhibition differ up to 20% from control, the higher score of 5 was assigned (low environmental impact); whenever growth inhibition differs 80% from control, the lower score was assigned (high environmental impact). For the mortality of *D. magna*, higher scores were assigned when neonates survival rate was above 80%, and lower scores were assigned when neonates survival rates were less than 20%. For the LOE AAMBI, the higher score was assigned to AAMBI values classified as "excellent (>121) and the lowest score was assigned when AAMBI values were classified as "bad" (<35).

Table S2. Parameters and the respective scores used to calculate the integrated index that includes the four LOE of freshwater parameters (Physicochemical, Metal concentrations, Macroinvertebrates and Toxicity) assessed in the study area.

	Ranking Categories	Score
Physico-chemical parameters	1)Physico-chemical values exceed limit permissible	
	Values are less than 50 times low of the limit permissible value	5
	Values are between 50 times low of the limit permissible value until the limit permissible value	3.6–4.9
	Values are from limit permissible value until 50 times upper of the limit permissible value	1.8–3.5
	Values are between 50 to 100 times upper the limit permissible value	0.1–1.7
	Values are more than 100 times upper of the limit permissible value	0
	2)Number of physicochemical parameters exceed limit permissible	
	Of the total amount of parameters, less than 19% of parameters exceed limit permissible	4.1–5
	Of the total amount of parameters, between 39% to 20% parameters exceed limit permissible	3.1–4.0
	Of the total amount of parameters, between 59% to 40% parameters exceed limit permissible	2.1–3.0
	Of the total amount of parameters, between 79% to 60% parameters exceed limit permissible	1.1–2.0
	Of the total amount of parameters, more than 80% of parameters exceed limit permissible	0–1.0
Metal concentrations	3) Metal concentration exceed limit permissible	
	Values are less than 50 times low of the limit permissible value	5
	Values are between 50 times low of the limit permissible value until the limit permissible value	3.6–4.9
	Values are from limit permissible value until 50 times upper of the limit permissible value	1.8–3.5
	Values are between 50 to 100 times upper the limit permissible value	0.1–1.7
	Values are more than 100 times upper of the limit permissible value	0
	4)Number of Metal species exceeding permissible limits	
	Of the total amount of metal species, less than 19% exceed limit permissible	4.1–5
	Of the total amount of metal species, between 39% to 20% exceed limit permissible	3.1–4.0
	Of the total amount of metal species, between 59% to 40% exceed limit permissible	2.1–3.0
	Of the total amount of metal species, between 79% to 60% exceed limit permissible	1.1–2.0

	Of the total amount of metal species, more than 80% exceed limit permissible	0–1.0
Toxicity bioassay	5) *Daphnia magna*	
	Neonates survive more than 80%	4.1–5
	Neonates survive between 60% and 79%	3.1–4.0
	Neonates survive between 40% and 59%	2.1–3.0
	Neonates survive between 20% and 39%	1.1–2.0
	Neonates survive less than 19%	0–1.0
	6) Phytotoxicity (*Lactuca sativa*)	
	20% of growth enhanced or inhibition	4.1–5
	21% to 40% of growth enhanced or inhibition	3.1–4.0
	41% to 60% of growth enhanced or inhibition	2.1–3.0
	61% to 80% of growth enhanced orinhibition	1.1–2.0
	More than 80% of growth enhanced or inhibition	0–1.0
Macroinvertebrates	7) AAMBI	
	Excellent (value <121)	5
	Very Good (range value 90–120)	3.6–4.9
	God (range value 50–89)	2.0–3.5
	Regular (range value 36–49)	1.4–1.9
	Bad (<35)	0–1.3

Table S3. Actual threshold values of CCME short- and long-term values calculated for the metals Cd, Zn, Mn and Cu. Calculations are based on pH, DOC and Hardness, as indicated in the equations found at the CCME Canadian Environmental Quality Guidelines (Vol. 2) 2002,

Site	pH	DOC	Hardness	Cd		Zn		Mn		Cu
				Short-term	Long-term	Short-term	Long-term	Short-term	Long-term	Long-term
P1	6.91	4.31	11.8	0.24	0.03	18.8	6.8	1019		<0.2
P2	6.7	1.85	3.9	0.08	0.01	6.1	2.0	387		
P3	6.55	2.4	5.3	0.11	0.01	8.4	3.4	506		
P4	6.67	3.86	7.6	0.15	0.02	12.7	5.2	696		
P5	7.17	2.98	9.0	0.18	0.02	13.7	3.7	805		0.615
P6	6.8	3.52	12.3	0.25	0.03	18.5	7.1	1059		<0.2
P7	6.61	4.07	37.9	0.78	0.07	48.9	25.5	2840	330	0.225
P8	8.06	1.89	34.5	0.71	0.07	37.6	5.3	2614	270	2.03
P9	7.18	1.74	23.4	0.48	0.05	26.7	7.2	1860	380	0.418
P10	7.37	2.27	37.4	0.77	0.07	42.1	10.7	2808	350	0.475
P11	7.79	2.39	30.6	0.63	0.06	36.0	6.4	2351	320	1.11
